# Quantification of B16 Melanoma Cells in Lungs Using Triplex Q-PCR - A New Approach to Evaluate Melanoma Cell Metastasis and Tumor Control

**DOI:** 10.1371/journal.pone.0087831

**Published:** 2014-01-31

**Authors:** Maria R. Sorensen, Sara R. Pedersen, Annika Lindkvist, Jan P. Christensen, Allan R. Thomsen

**Affiliations:** Department of International Health, Immunology and Microbiology, University of Copenhagen, Copenhagen, Denmark; University of Tennessee, United States of America

## Abstract

Skin cancer is the most common type of all cancers. However, it comprises several different types of cancers, one of which is malignant melanoma. Even though melanomas only make up about 5% of skin cancers, they are responsible for the majority of skin cancer deaths due to the poor chance of survival once the tumor has metastasized. In the present study, we have developed a new assay for quantitative analysis of B16 melanoma metastasis in the lungs. We have used a triplex Q-PCR to determine the expression of the melanoma genes GP100/Pmel and tyrosinase-related protein 2 (TRP-2), and found that B16.F10gp cells were detectable in the lungs as early as 2 hours after intravenous challenge with ≥10^4^ tumor cells. When investigating the gene expression as a function of time, we observed a gradual decrease from 2–24 hours post tumor challenge followed by an increase of approximately 2 log_10_ on day 11. The early decrease was accelerated in the presence of activated NK cells. To further evaluate our assay, we also investigated the level of metastasis in the context of vaccination with replication defective adenoviral vectors, Ad-Ii-GP and Ad-GP, previously found to significantly delay the outgrowth of subcutaneous melanomas. Results obtained using Q-PCR were compared to conventional counting of metastatic foci under a dissection microscope. A marked reduction in gene expression was observed in the lungs after vaccination with both vectors; however, Ad-Ii-GP showed the highest protection, and matching results were obtained by enumeration of visible tumor nodules on the lung surfaces. Finally, we could show that inhibition of tumor metastasis required antigen-specific CD8 T cells and IFNγ, but not perforin. In conclusion, the presented results validate triplex Q-PCR as a fast, objective, and quantitative method for analysis of melanoma metastasis in the lungs.

## Introduction

According to the World Health Organization, about 132,000 new cases of malignant melanoma are diagnosed globally each year and, notably, this number seems to be increasing [1]. Since primary melanomas often are diagnosed early during tumor development, the overall survival rate of patients with malignant melanoma is approximately 90% [2]. Unfortunately, treatment of metastatic melanomas has proven very difficult, and while the 5-year survival rate for patients with localized melanomas is as high as 98%, only about 15% of patients diagnosed with distant metastases survive the first 5 years [2]. Metastasis is therefore the leading cause of melanoma-related deaths, and even though melanomas represent less than 5% of all skin cancers, they are responsible for the large majority of skin cancer-related deaths [3].

Today, treatments of malignant melanoma include surgery, chemotherapy, immunotherapy, and radiation therapy; however, new approaches and cancer therapies are needed in order to raise the life expectancy of patients with metastatic disease [3]. At present, numerous new treatment strategies against melanoma have been evaluated, and several are still under investigation. Often, murine models are used as a first stage in the evaluation of potential new treatments, and the murine melanoma cell line B16.F10 is widely used in experimental tumor immunology and in the testing of new anti-cancer vaccine strategies. The B16.F10 descends from the B16 cell line which was established from a spontaneous melanoma in the ear of a C57BL/6 mouse in 1954 [4]. In the early 1970s, Fidler *et al.* created the B16.F10 cell line by repeatedly selecting B16 cells with a high lung colonization capacity [5]. Intravenous (i.v.) inoculation with B16.F10 cells therefore leads to the formation of lung metastases.

Up until now, studies aiming to determine the level of metastasis of the lungs have primarily relied on visual inspection by counting the number of melanoma nodules, either on the surface of the lungs or on cross-sections [6]–[10]. However, this method is time consuming, tedious, only semi-quantitative, and furthermore requires blinded counting to avoid biased results. For these reasons, we have developed a high-throughput, quantitative, and objective method for the quantification of B16 melanoma metastases in the lungs and potentially other organs with a low background of melanocytes. Here, we determine the level of B16.F10gp metastasis to the lungs using a triplex Q-PCR method, which detects the expression of the melanoma genes GP100 and tyrosinase-related protein 2 (TRP-2) relative to the reference gene glyceraldehyde-3-phosphate dehydrogenase (GAPDH). The B16.F10gp cells differ from the original B16.F10 cells in that they have been genetically modified to express the dominant MHC class I-restricted epitope GP_33–41_ of the lymphocytic choriomeningitis virus (LCMV) glycoprotein GP [11], making the cells useful as in vivo targets for tumor immunotherapy (see further below). In addition, we have also tested a one-step triplex Q-PCR method in the study to further speed-up the melanoma detection assay. In this case, the cDNA synthesis step is included in the Q-PCR reaction, and each sample, therefore, only has to be handled once after RNA extraction.

To further validate the practical use of the triplex Q-PCR method in a B16 tumor setting, we compared levels of metastasis as determined by visual inspection or by expression of GP100 and TRP-2 in mice vaccinated with replication-defective adenoviral vectors encoding either GP tethered to invariant chain (Ii) or GP alone [12]. We have previously used these vectors as vaccination tools in a solid B16.F10gp tumor model where we achieved complete protection against the growth of subcutaneous (s.c.) B16.F10gp melanomas after prophylactic vaccination with the Ad-Ii-GP vector [13].

In this study, we demonstrate that the triplex Q-PCR method is a new, sensitive, and easy-to-perform assay to objectively quantify metastasis in the lungs after challenge with B16 cells, and hopefully, its application will aid in accelerating the testing of new anti-cancer drugs and vaccine candidates in the future.

## Materials and Methods

### Ethical statement

Experiments were conducted in accordance with national Danish guidelines (Amendment # 1306 of November 23, 2007) regarding animal experiments as approved by the Danish Animal Experiments Inspectorate, Ministry of Justice, permission number 211/5187.

### Mice

Female C57BL/6 mice were purchased from Taconic M&B (Ry, Denmark). Mice deficient in perforin and/or IFNγ were the progeny of breeding pairs originally obtained from The Jackson Laboratory (Bar Habor, ME, USA). All mice were allowed to acclimatize for at least one week before entering into experiments; mice entered experiments when 7–10 weeks old. The experimental procedures were approved by the national ethics committee on experimental animal welfare and performed according to national guidelines.

### Cell material and tumor inoculation

In house melanoma cell lines B16.F10 and B16.F10gp were kind gifts from H. Pircher (University of Freiburg, Germany) [5], [11]. These two cell lines are genetically identical with the exception of a modification in B16.F10gp, which results in the expression of the dominant LCMV epitope GP_33-41_. Additionally, B16.F10 cells were obtained directly from ATCC (CRL-6475). Tumor cells were propagated in DMEM 1965 supplemented with 10% FBS, as well as 1% L-glutamine, penicillin, and streptomycin. For the B16.F10gp cell line, 0.6 mg/ml of Geneticin G-418 sulphate neomycin was also included in the culture medium. The mouse lymphoma cell line EL-4 from ATCC (TIB-39) was cultured in RPMI 1640 supplemented with 10% FBS and 1% L-glutamine, 2-mercaptoethanol, penicillin, and streptomycin. Total RNA from the amelanotic cell line B78-D14 was kindly provided by Peter Holst, this institute.

For tumor inoculation, mice were injected with 10^2^–10^6^ B16.F10gp cells into the tail vein. For counting of lung metastases, lungs were harvested and put into 4% PFA. On the same day, the lobes of the lungs were gently unfolded and metastases visible on the lung surfaces were enumerated using a dissection microscope. Each sample was scored by 2 or 3 blinded observers, and averages for individual animals were depicted.

### Viral vectors and vaccination

Ii and either LCMV GP or chicken ovalbumin (OVA) were amplified and fused by overlapping PCR as described previously [12]. The Ii plasmid was a kind gift from N. Koch (University of Bonn, Bonn, Germany) and has previously been described [14]. Human adenovirus 5 variants were produced through homologous recombination using standard methods, and the inserts were introduced into the E1 gene under a CMV promoter [15]. After purification, adenoviral stocks were immediately aliquoted and frozen at −80C° in 10% glycerol, and the infectivity of adenovirus stocks was determined using the Adeno-X Rapid Titer Kit (Clontech). For vaccination, mice were inoculated s.c. in the right hind footpad with 2×10^7^ IFU of Ad-GP, Ad-Ii-GP, or Ad-Ii-OVA. Vesicular Stomatitis Virus (VSV) of the Indian strain was produced, stored, and quantified as previously described [16]. Mice were infected i.v. with 10^6^ PFU VSV, which is a nonlethal dose for immunocompetent mice.

### Antibodies

Anti-CD8 mAb from YTS-156 and YTS-169 hybridoma cells (a kind gift from S Cobbold, University of Oxford) was purified on protein G columns, and CD8 T cells were depleted by intraperitoneal injection with 100 µg of mAb from each clone one day before and one day after vaccination and tumor challenge. Depletion of NK cells was achieved by intraperitoneal administration of 100 µg of anti-NK1.1 mAb one day prior to tumor challenge. The anti-NK1.1 mAb was produced by PK136 hybridoma cells (ATCC HB-191) and purified on a protein G column. The efficiency of antibody mediated cell depletion was ascertained by flow cytometry.

### RNA extraction and cDNA synthesis

Total RNA was extracted using RNeasy Midi Kit (Qiagen, cat. #75144) as described in the following. Mice were euthanized and lungs, liver, and lymph nodes were isolated and snap frozen in liquid nitrogen before being stored at −80°C. For cell cultures, cells were harvested in a good growth phase and 10^7^ cells were pelleted. Organs or cell pellets were mixed with RTL buffer and homogenized using an IKA T10 basic ULTRA-TURRAX homogenizer (Bie & Berntsen). The blender head was washed with 3× PBS, 1× TAE buffer, 1× 70% EtOH, and 1× 96% EtOH between every sample. Samples were then centrifuged for 5 min at 6000 rpm and the supernatant was isolated. For homogenized organs, this step was repeated. Equal volumes of 70% EtOH were added to the supernatant, samples were vortexed, and half of the volume of the samples was added to RNeasy Midi spin columns and centrifuged for 10 min at 5000 rpm. The step was repeated with the last half of the samples. The columns were then washed once in RW1 buffer followed by centrifugation at 5000 rpm for 5 min, and twice in RPE buffer succeeded by centrifugation at 5000 rpm for 2 and 5 min, respectively. The columns were then transferred to new tubes and RNAse free H_2_O was added to the spin columns. The samples were left to stand for 1 min and then centrifuged at 5000 rpm for 3 min. This step was repeated and total RNA was collected and stored at −80°C. Subsequently, total RNA concentration and purity was measured using a NanoDrop 2000c Spectrophotometer (Thermo Scientific) and the associated computer program NanoDrop 2000.

Finally, cDNA synthesis was performed with 1 µg total RNA per sample using a RevertAid™ First Strand cDNA Synthesis Kit from Fermentas (Cat. #K1622). Briefly, total RNA was mixed with DEPC-treated water and then 10× Reaction buffer with MgCl_2_ as well as DNAse I was added to remove any DNA. Next, EDTA was added to inactivate the DNAse, followed by the addition of Oligo dT-primer and then 5× reaction buffer, dNTP, and ribonuclease inhibitor. Subsequently, RevertAid H-minus M-MµLV reverse transcriptase (RT) was added to one of two duplicates of each sample. Lastly, samples with RT and without RT were run in a GeneAmp PCR system 9700 machine (Applied Biosystems) at the following temperatures: 42°C for 60 min, 70°C for 10 min, and 4°C until collected. Samples were stored at 4°C.

### Quantitative real time polymerase chain reaction (Q-PCR)

Singleplex and triplex Q-PCRs were performed according to the manufacturer's guidelines using Brilliant II QPCR Master Mix (Stratagene #600804), and Q-PCRs were run using the following program: 95°C/10 min+40×(95°C/30 s+60°C/1 min+72°C/30 s).

One-step triplex Q-PCR was performed with 0.1 µg DNAse treated RNA per sample using the Brilliant II QRT-PCR Master Mix Kit, 1-step from Stratagene (Cat. #600809). The Q-PCR program was: 50°C/30 min+95°C/10 min+40×(95°C/30 s+60°C/1 min+72°C/30 s).

Primers and probes were used: GP100 forward: 5′ AGC ACC TGG AAC CAC ATC TA 3′ (200 nM), GP100 reverse: 5′ CCA GAG GGC GTT TGT GTA GT 3′ (200 nM), GP100 probe: 5′ Hex-CAC TAC AAA AGT TGT GGG TAC TAC ACC TG-BHQ-1-3′ (300 nM), TRP-2 forward: 5′ TTA GGT CCA GGA CGC CCC 3′ (200 nM), TRP-2 reverse: 5′ CTG TGC CAC GTG ACA AAG GC 3′ (200 nM), TRP-2 probe: 5′ Fam-AAG GCC ATT GAT TTC TCT CAC CAA GGG CC-BHQ-1-3′ (300 nM), GAPDH forward: 5′ CAA TGT GTC CGT CGT GGA 3′ (200 nM), GAPDH reverse: 5′ GAT GCC TGC TTC ACC ACC 3′ (200 nM), GAPDH probe: 5′ Cy5-CGC CTG GAG AAA CCT GCC AAG TAT-BHQ-1-3′ (200 nM).

Each sample was run in triplicate together with a No RT control, in which no reverse transcriptase was added during the cDNA synthesis. In addition, a standard curve was run in duplicate. The Q-PCR data analysis was performed on a Mx3005P Real-time Q-PCR instrument, and the results were analysed using the software programme MxPro – Mx3005p v. 4.1, Stratagene.

## Results

### Validation of the triplex Q-PCR

Three experimental setups were used for assaying the expression of the melanoma-specific genes GP100 and TRP-2. One setup consisted of three singleplex Q-PCRs run in parallel, each detecting one of these genes or the reference gene GAPDH. The second was a triplex Q-PCR in which both target genes and the reference gene were amplified together. The third was a one-step triplex Q-PCR, which differed from the triplex in that the cDNA synthesis step was included in the Q-PCR reaction. Examination of standard curves and amplification plots to evaluate the quality of the primers and probes, as well as the fit of the standard curve to the plotted standard data points, revealed that the amplification efficiencies ranged from 90.9-113.2% and the RSq values varied between 0.982–0.999. From this we concluded that all three Q-PCRs performed satisfactorily (Fig. S1A–C).

**Figure 1:**
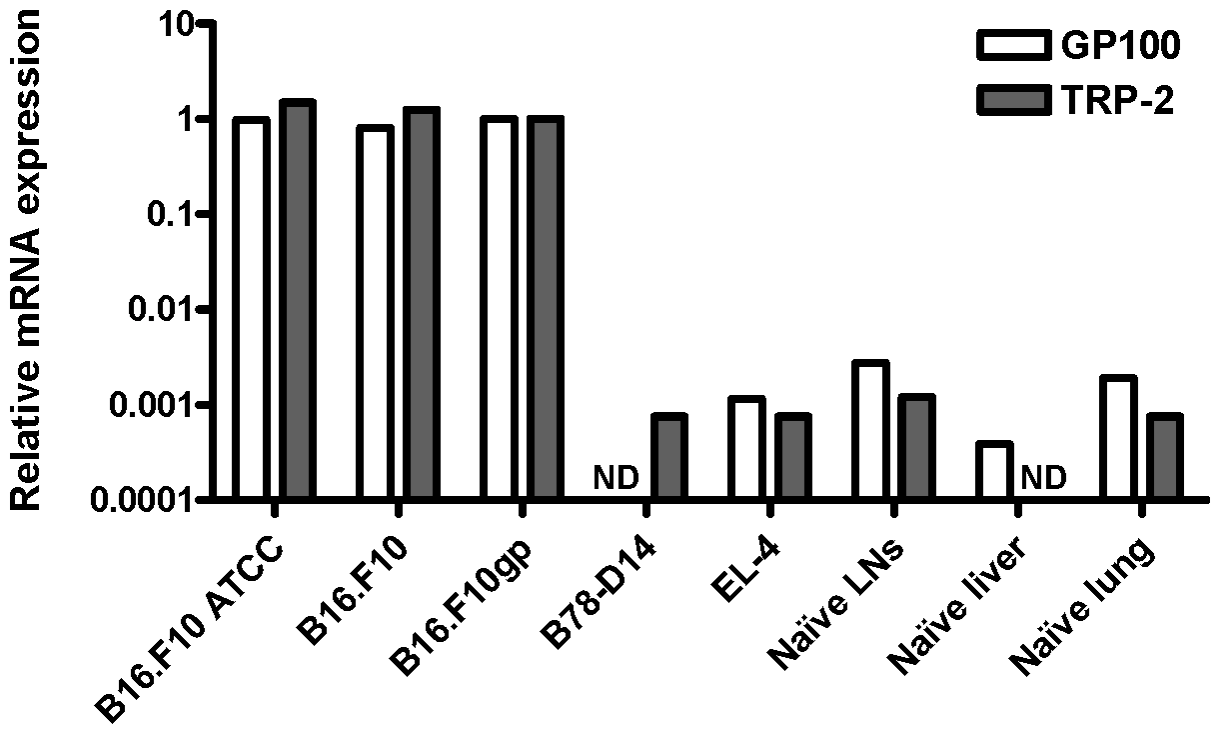
Relative mRNA expression of melanoma genes GP100 and TRP-2 in different cell lines and normal tissues. To compare the expression of GP100 and TRP-2 in melanoma cells to that in other cell types, total RNA was extracted from three different sublines of B16.F10 melanoma cells, including a variant obtained directly from ATCC, from the amelanotic melanoma cell line B78-D14, and from the lymphoma cell line EL-4 (to serve as negative control). Using Q-PCR, gene expression was compared to that in three different tissues from normal C57BL/6 mice: liver, lungs and lymph nodes (LNs). Melanoma gene expression was quantified relative to the housekeeping gene GAPDH. The relative mRNA expression of B16.F10gp cells was used as calibrator and set to 1.

In order for the Q-PCR approach to be relevant as an assay for detection of melanoma metastasis, it requires that TRP-2 and GP100 are highly expressed in commonly applied melanoma cell lines, but not to any substantial degree in normal tissues commonly subject to melanoma metastasis. To test this, we made cDNA from RNA of three different sublines of B16.F10, including a variant obtained directly from ATCC; from the amelanotic melanoma cell line B78-D14; and from the lymphoma cell line EL-4 (to serve as negative control). Gene expression was compared to that in three different tissues from normal C57BL/6 mice: liver, lung and lymph nodes (Fig. 1). Using the triplex Q-PCR, we observed very substantial and about equally high expression of TRP-2 and GP100 in all melanotic melanoma cell lines regardless of origin. In contrast, amelanotic melanoma cells were negative (cf. EL-4 lymphoma cells), and very low if any expression was detected in relevant normal tissues.

**Figure 2:**
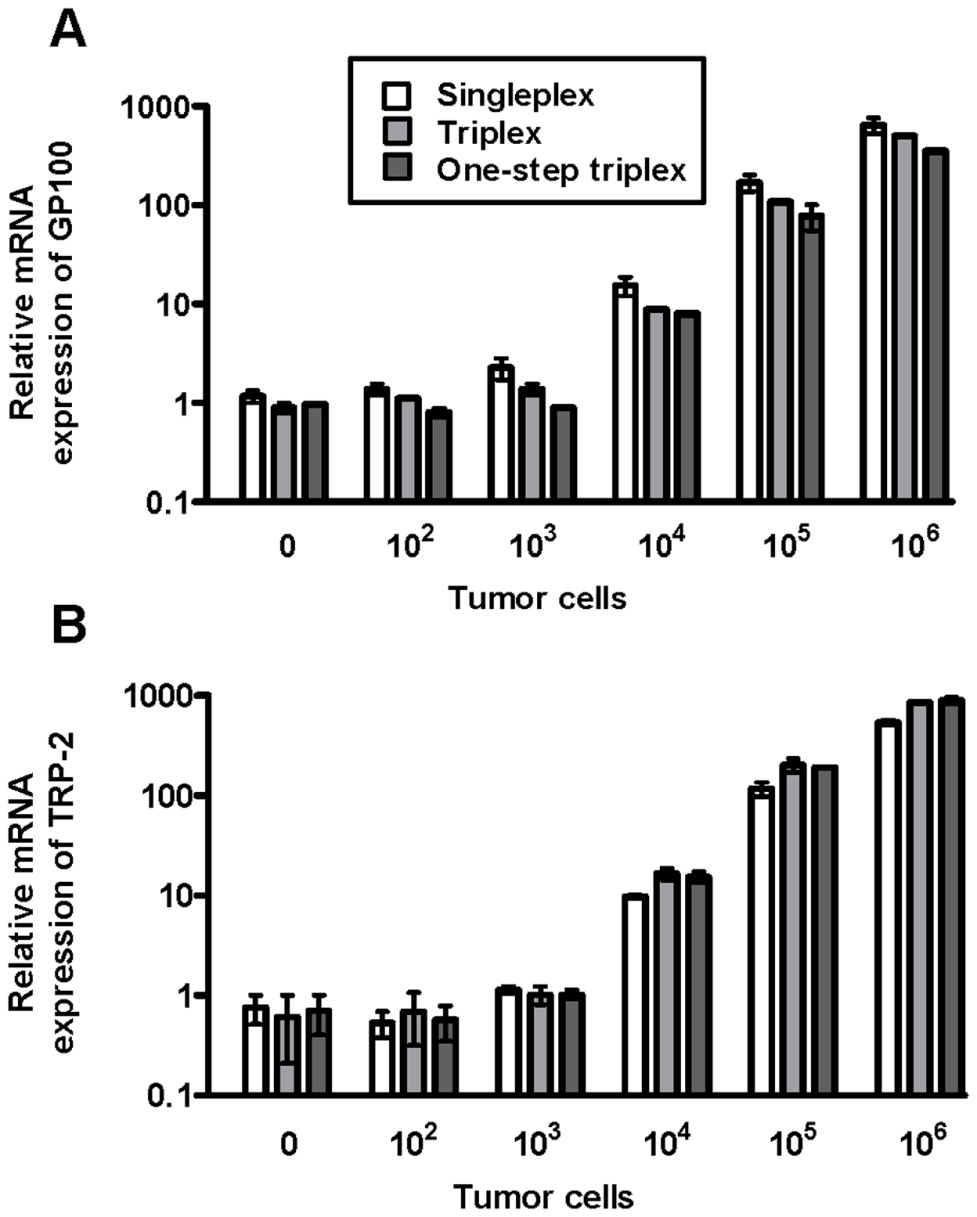
Relative mRNA expression of melanoma genes in lungs as a function of tumor cell dose. Mice were injected with 10^2^, 10^3^, 10^4^, 10^5^, or 10^6^ B16.F10gp melanoma cells into the tail vein. Two hours later, lungs were collected and snap frozen in liquid nitrogen. Total RNA was extracted and either used directly for one-step triplex Q-PCR (dark grey) or converted into cDNA and used for singleplex (white) or triplex Q-PCR (light grey). The mRNA expression of melanoma genes GP100 (A) and TRP-2 (B) was quantified relative to the house keeping gene GAPDH. The experiment was performed twice with similar outcomes, and bars depict medians and ranges of two mice per group. The relative mRNA expression of one untreated mouse was used as the calibrator and set to 1.

Next, we compared the accuracy and detection limit of these Q-PCRs when it came to evaluating lung metastasis. For this purpose, C57BL/6 mice were injected i.v. with ten-fold dilutions of B16.F10gp melanoma cells (10^2^–10^6^) and 2 hours later, lungs were removed, and RNA was extracted and either used directly in a one-step triplex Q-PCR or converted into cDNA and used in the singleplex and triplex Q-PCRs. As seen in Fig. 2, target gene expression exceeded background levels when mice had been injected with at least 10^4^ melanoma cells, and the expression of both GP100 and TRP-2 was linear on a logarithmic scale, as expected when using 10× dilutions of the injected melanoma cells. This correlation was observed for the singleplex, triplex, and one-step triplex Q-PCR, which all showed similar expression patterns. For future experiments, we decided to use the triplex Q-PCR in combination with an inoculum of 10^5^ B16.F10gp cells, as this resulted in easily detectable gene expression levels in lungs shortly after tumor inoculation (see Fig. 2).

### Timeframe of the detection of melanoma metastasis by triplex Q-PCR

**Figure 3:**
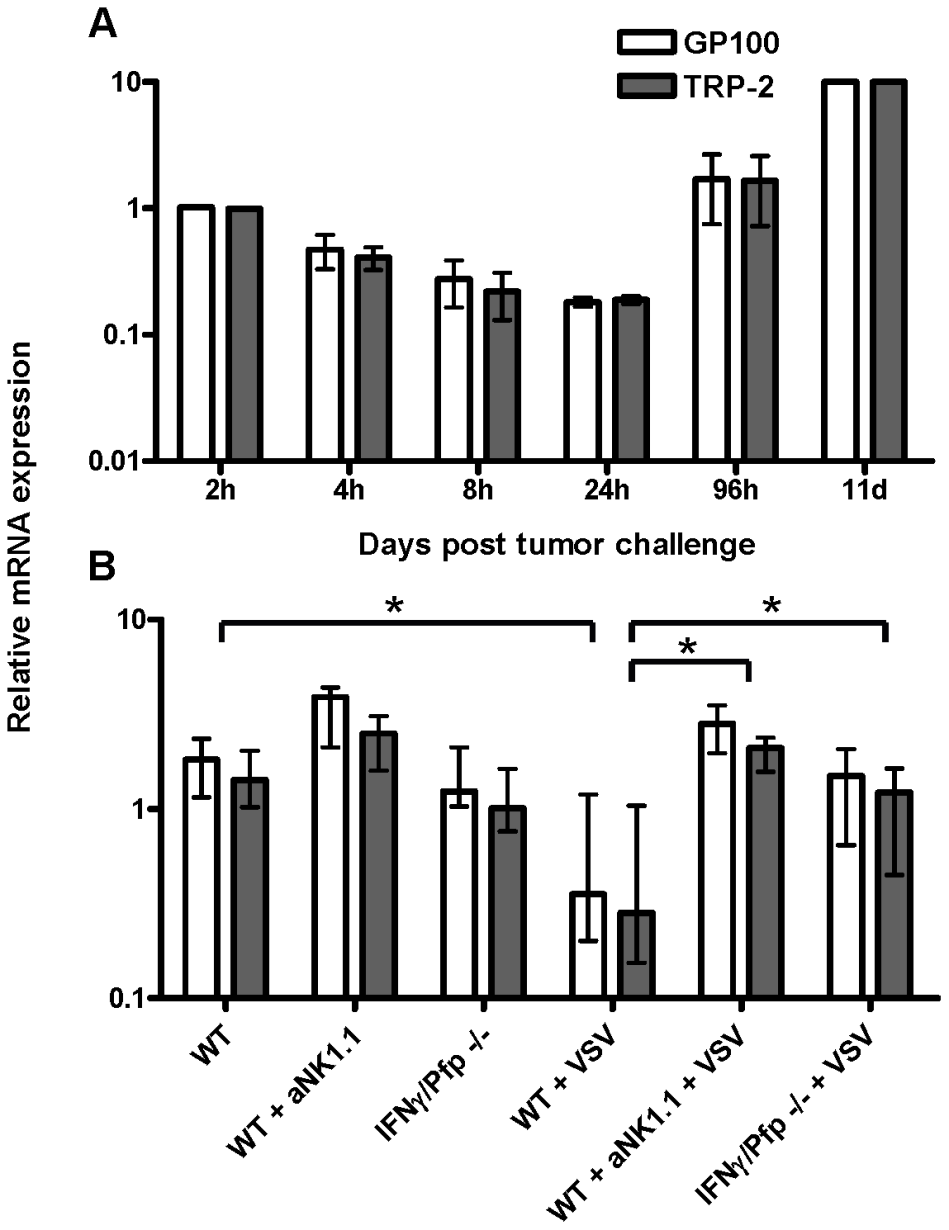
Relative mRNA expression of melanoma genes as a function of time after i.v. tumor inoculation. (A) Mice were injected i.v. with 10^5^ B16.F10gp melanoma cells and lungs were collected 2, 4, 8, 24, 96 hours or 11 days later and snap frozen in liquid nitrogen. Total RNA was extracted and converted into cDNA and the expression of GP100 (white) and TRP-2 (grey) was detected using triplex Q-PCR. The experiment was performed twice with similar outcomes. (B) Mice were infected i.v. with 10^6^ pfu VSV or left untreated. One day later, depleting anti-NK1.1 antibodies were administered i.p. to some groups and on the next day, all mice were injected i.v. with 10^5^ B16.F10gp cells. Lungs were collected 4 hours after tumor inoculation and the relative mRNA expression of GP100 (white) and TRP-2 (grey) was obtained using triplex Q-PCR analysis. The relative mRNA expression in the lungs of one mouse collected 2 hours after tumor inoculation, and of one mouse vaccinated with Ad-Ii-OVA and lungs isolated 6 days after tumor challenge (see Fig. 5B) were used as calibrators in A and B, respectively, and set to 1. Shown are medians and ranges of 5 mice per group; results are representative of two experiments. WT =  wild type, IFNγ =  interferon gamma deficient, Pfp =  perforin deficient, * denotes p<0.05.

To establish the time-frame for analyzing tumor control in the lungs, we next studied the expression of the melanoma genes as a function of time after tumor injection. Lungs were collected 2, 4, 8, 24, 96 hours or 11 days post tumor inoculation. A gradual decrease in gene expression was observed between 2–24 hours, followed by a marked increase, which on day 11 post challenge reached a level of about 100 times the lowest point (Fig. 3A).

We reasoned that NK cells might impact the early phase of tumor establishment in the lungs and that the early decrease in gene expression could reflect such activity [17], [18]. We tested this hypothesis by removing NK cells with depleting anti-NK1.1 antibodies as well as by increasing their activity through the infection with VSV, a known inducer of type I IFNs and NK cell activation [19], [20]. Depletion of NK cells did not markedly increase the level of melanoma gene expression compared with untreated controls, however, a tendency towards an elevated gene expression was observed (Fig. 3B). On the other hand, GP100 and TRP-2 gene expression levels in the lungs of VSV infected mice were clearly lower than those in uninfected controls. Treatment with NK cell depleting antibodies reversed this effect.

In addition, we also investigated whether IFNγ and/or perforin played a role in the early drop in GP100 and TRP-2 gene expression. No difference was observed in gene expression when comparing uninfected IFNγ or perforin deficient mice to matched wild type (WT) mice (data not shown), and the same was true for IFNγ/perforin deficient mice (Fig. 3B). However, VSV-infected IFNγ/perforin deficient mice failed to present the VSV-associated decrease in the gene expression, which was observed in infected WT mice (Fig. 3B). Taken together, these results are consistent with the view that activated NK cells play a role in controlling the early phase of tumor metastasis in the lungs and suggest a role for IFNγ and/or perforin during VSV-induced reduction of metastasis.

### Vaccination with recombinant adenoviral vectors reduces tumor gene expression

**Figure 4:**
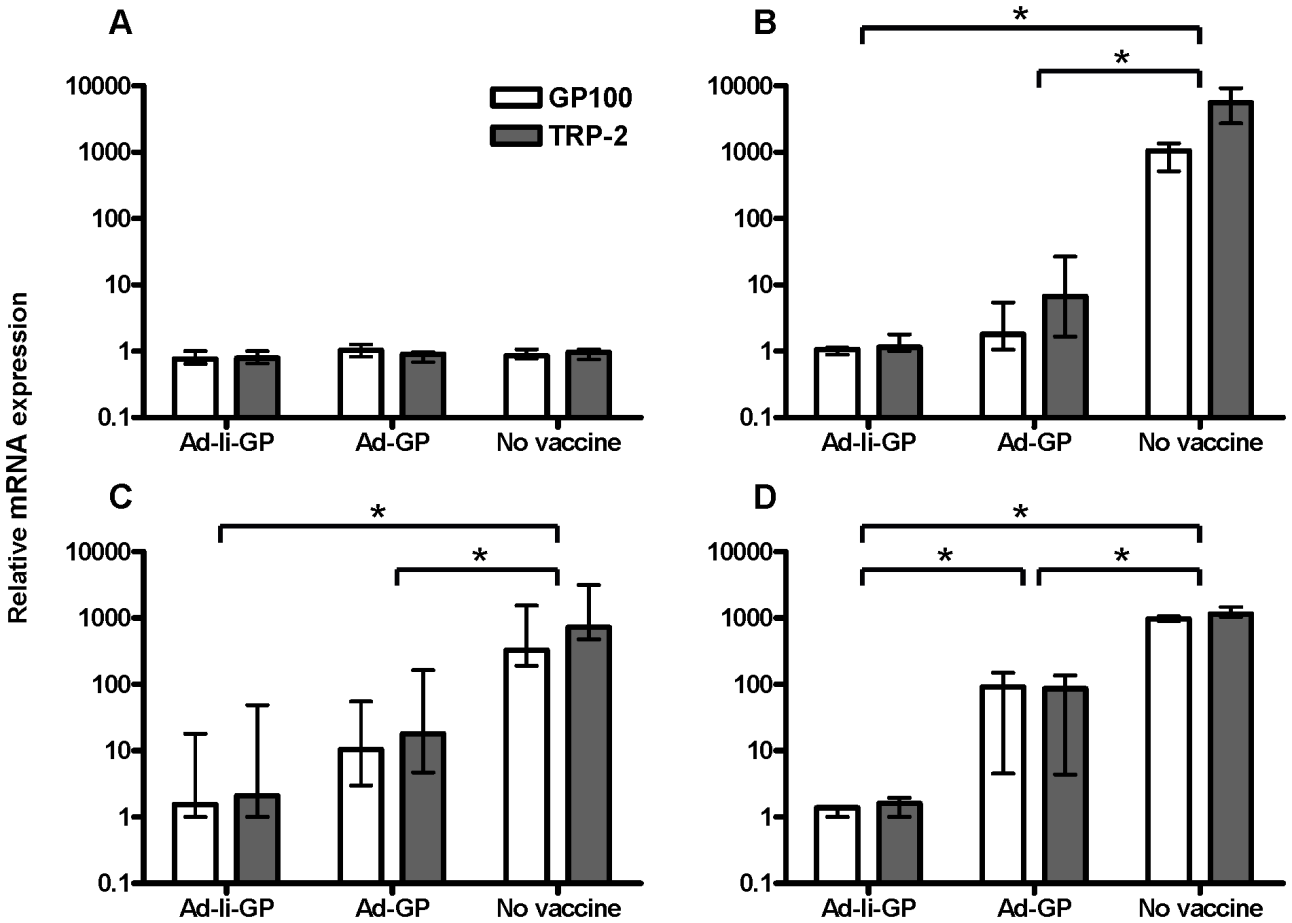
Reduced melanoma lung metastasis after vaccination with adenoviral vectors Ad-GP and Ad-Ii-GP. Mice were vaccinated with 2×10^7^ IFU Ad-Ii-GP, Ad-GP, or left unvaccinated and subsequently inoculated i.v. with 10^5^ B16.F10gp melanoma cells either on the same day (D), 11 (A and B) or 31 (C) days later. Lungs were collected for triplex Q-PCR analysis either 2 hours (A) or 11 days (B, C and D) after tumor inoculation. Shown are medians and ranges of the relative mRNA expression of GP100 (white) and TRP-2 (grey) for groups of n = 3–4 mice. Each experiment was performed one or two times, and the relative mRNA expression in the lungs of one mouse from the group vaccinated with Ad-Ii-GP was used as the calibrator and set to 1. Ad =  adenoviral vector, Ii =  invariant chain, GP =  LCMV glycoprotein GP, * denotes p<0.05.

To further validate the use of the triplex Q-PCR as a method for detecting B16 lung metastasis, we next studied the inhibition of tumor gene expression induced by vaccination with replication-deficient adenoviral vectors. As mentioned previously, the Ad-GP and Ad-Ii-GP vaccines have both been shown to induce clear protection against subsequent s.c. challenge with B16.F10gp tumor cells; however, the Ii tethered vaccine worked much more efficiently than the conventional vaccine in a therapeutic setting [13]. To test these vaccines in the metastasis model, mice were vaccinated with Ad-Ii-GP, Ad-GP, or left untreated. When tumor cells were administered 11 days post vaccination and lungs were isolated 2 hours after tumor inoculation, the level of gene expression was the same in all the groups (Fig. 4A). This is despite the fact that the activity of GP-specific CD8 T cells is maximal around day 10–14 after vaccination with the Ad-Ii-GP vaccine [12], [21]. On the other hand, when mice were given tumor cells 11 or 31 days post vaccination, and lungs were collected 11 days post tumor inoculation, a clear reduction in gene expression was detected in mice vaccinated with Ad-Ii-GP or Ad-GP compared to controls (Fig. 4B–C). Finally, we examined the impact of vaccinating mice on the day of tumor challenge; again tumor gene expression was determined 11 days after challenge. In this case, we found that gene expression was markedly suppressed in Ad-Ii-GP vaccinated mice compared to controls (Fig. 4D). This suppression was also present in Ad-GP vaccinated mice, albeit to a lesser extent. Taken together, these data indicated that prophylactic vaccination with Ad-Ii-GP or Ad-GP is able to reduce tumor gene expression in the lungs. Even vaccination at the time of tumor challenge clearly impacted the expression of both tumor genes, particularly when using the Ii tethered vaccine.

### Validation of the Q-PCR approach compared to conventional enumeration of metastases by visual inspection

While the above results strongly suggested that metastasis could be quantified by our Q-PCR, we lacked formal proof that suppression of gene expression correlated with immune mediated control of tumor metastasis. Consequently, we wanted to perform a head-to-head comparison of results obtained by tumor focus counting to matched results obtained by Q-PCR.

**Figure 5:**
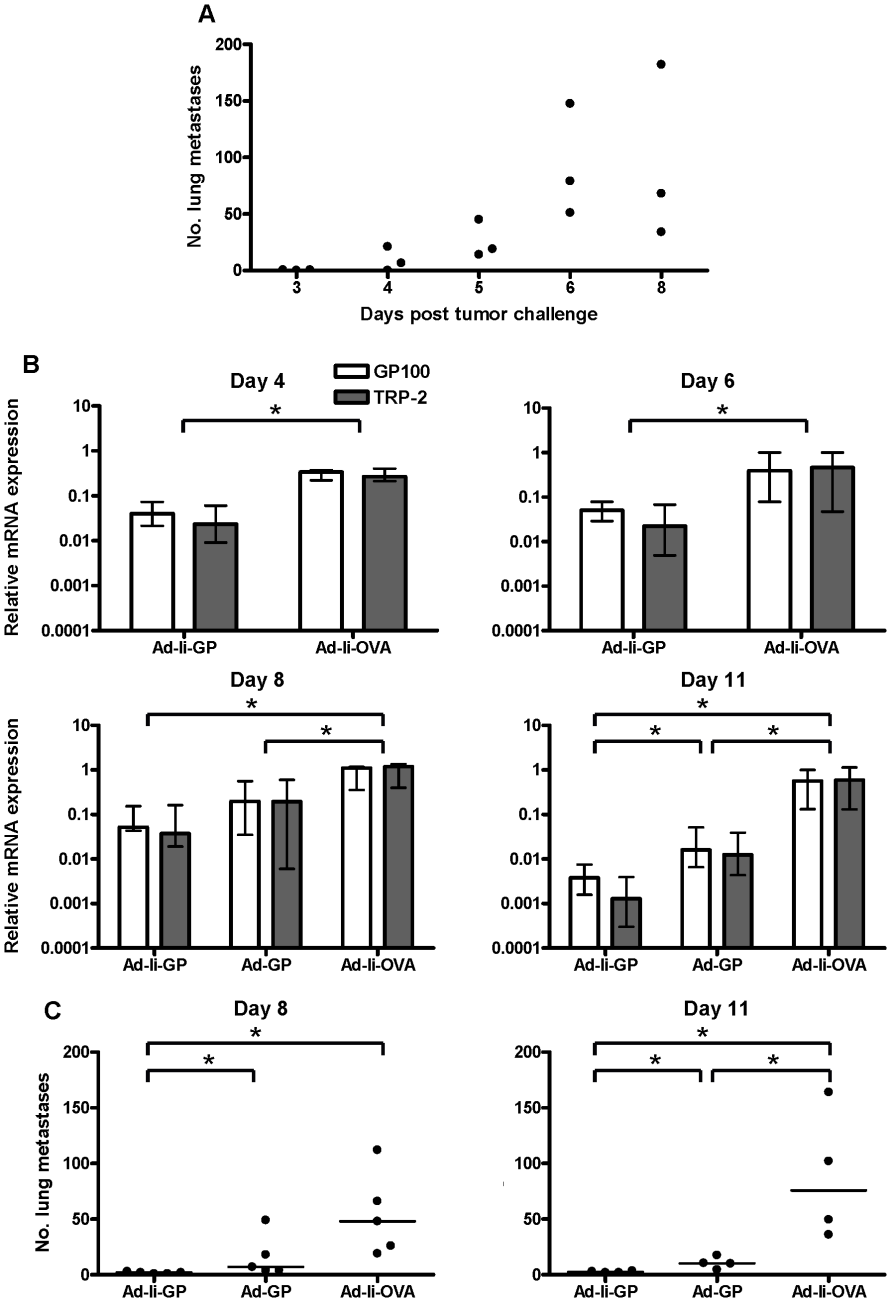
Comparison of lung metastasis as a function of time after tumor inoculation. (A) Naïve mice were inoculated i.v. with 10^5^ B16.F10gp melanoma cells, and on the indicated days metastatic foci were enumerated as described in M & M. Each point represents one mouse, and the experiment was performed twice with similar results. (B) Mice were vaccinated with 2×10^7^ IFU of the indicated adenoviral vectors and inoculated i.v. with 10^5^ B16.F10gp melanoma cells 14 days later. Lungs were collected for triplex Q-PCR analysis 4, 6, 8, or 11 days after tumor inoculation. Shown are medians and ranges of the relative mRNA expression of GP100 (white) and TRP-2 (grey) for groups of n = 4–5 mice. For days 4, 6 and 8, the relative mRNA expression of one mouse from day 6 vaccinated with Ad-Ii-OVA was used as the calibrator and set to 1, while for day 11, the relative mRNA expression of one mouse from day 11 vaccinated with Ad-Ii-OVA was used as the calibrator and set to 1. Ad =  adenoviral vector, Ii =  invariant chain, GP =  LCMV glycoprotein GP, OVA =  ovalbumin. (C) Enumeration of metastatic foci by visual inspection of lungs from mice challenged in parallel to those in part B; each point represents one mouse, and the experiment was performed twice with similar results. * denotes p<0.05.

To this end, we first wanted to determine the time required for objective quantification of lung metastatic foci after tumor inoculation. Naïve mice were injected with 10^5^ tumor cells i.v. and 3, 4, 5, 6 and 8 days later, mice were sacrificed and metastatic foci on the surface of the lungs were enumerated using a dissection microscope (Fig. 5A). While no foci were detected on day 3 post challenge, small spots could be detected by day 4. However, neither on this day nor on day 5 after challenge was it possible to count the number of melanoma foci completely objectively due to their small size. By day 6 post challenge, foci had reached a size that allowed for precise counting. This was supported by the finding that, while the size of individual foci continued to increase, numbers of detectable foci leveled off between days 6 and 8 after challenge.

In order to get an impression of how quickly the impact of an adaptive immune response on tumor growth could be detected by Q-PCR, tumor cells were injected into Ad-Ii-GP vaccinated mice at the height of the T cell response. Lungs were collected for triplex Q-PCR 4, 6, 8, or 11 days later and sham vaccinated mice served as controls. As can be seen in Fig. 5B, a significant impact of anti-tumor vaccination could be observed already 4 days after the tumor challenge, which is slightly earlier than it would be possible based on enumeration of visible foci (see above). However, the difference and thus the sensitivity of the tumor control increased with time, reaching between 2 and 3 orders of magnitude by day 11.

For a direct comparison between the methods, mice challenged in parallel were sacrificed and visible tumor foci were enumerated on days 8 and 11 after challenge (Fig. 5C). As can be seen from a comparison of Fig. 5, parts B and C, the overall pattern was clearly comparable irrespective of the assay method used to evaluate lung metastasis, thus supporting that inhibition of tumor gene expression appropriately reflected control of tumor metastasis. However, the separation between the groups tested tended to increase with time in terms of fold differences when using the Q-PCR approach.

### Identification of the effector mechanism underlying vaccine-induced tumor control in the lungs

**Figure 6:**
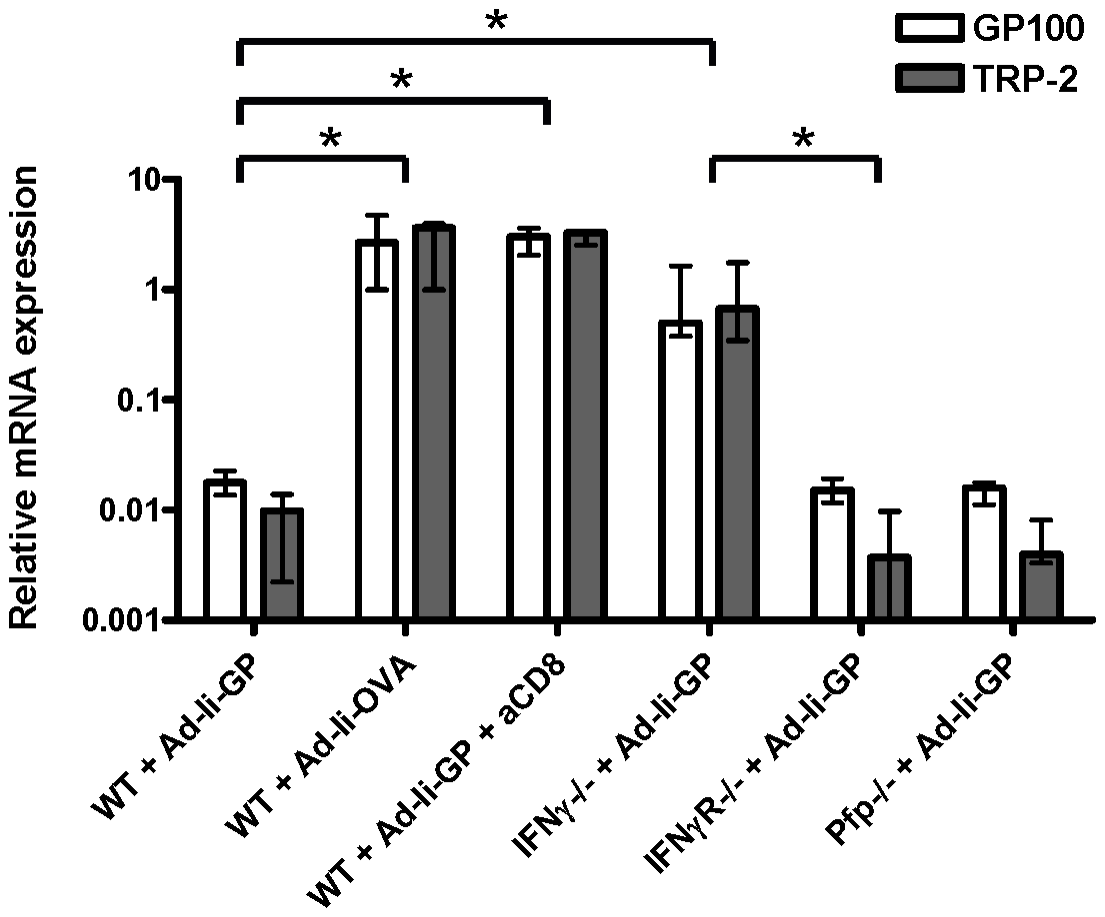
CD8 T cells and IFNγ are required for Ad-Ii-GP induced protection against melanoma lung metastasis. Mice were vaccinated with 2×10^7^ IFU Ad-Ii-GP or Ad-Ii-OVA and subsequently injected i.v. with 10^5^ B16.F10gp melanoma cells. Depleting anti-CD8 antibodies were administered i.p. one day before and one day after vaccination and tumor challenge. Lungs were collected 11 days post vaccination and tumor inoculation, and the relative mRNA expression of GP100 (white) and TRP-2 (grey) was detected by triplex Q-PCR. Bars show medians and ranges of 4–5 mice per group, and the relative mRNA expression in the lungs of one WT mouse vaccinated with Ad-Ii-OVA was used as the calibrator and set to 1. The experiment was performed twice with similar results. WT =  wild type, IFNγ =  interferon gamma deficient, IFNγR =  interferon gamma receptor deficient, Pfp =  perforin deficient, Ad =  adenoviral vector, Ii =  invariant chain, GP =  LCMV glycoprotein GP, OVA =  ovalbumin, * denotes p<0.05.

Finally, we wanted to determine the effector mechanism underlying the vaccine-induced inhibition of B16.F10gp lung metastasis. To this end, WT and relevant gene deficient (perforin, IFNγ, and IFNγR) mice were vaccinated and subsequently challenged with tumor cells; depleting anti-CD8 antibodies were administered one day before and one day after vaccination and tumor inoculation. As seen in Fig. 6, we observed that vaccinated WT mice treated with depleting anti-CD8 antibodies as well as mice deficient in IFNγ showed higher melanoma gene expression compared to untreated WT mice. In contrast, perforin and IFNγR deficient mice were able to reduce melanoma gene expression to the same degree as WT mice. Collectively, these results indicate that Ad-Ii-GP-induced reduction of melanoma metastasis is mediated by CD8 T cells and that IFNγ, but not perforin, inhibits tumor cell growth.

## Discussion

Q-PCR analysis is currently used in a number of fields including clinical diagnosis, forensic science, and molecular research [22]. Regardless of its application, a Q-PCR measures gene levels by detecting either DNA or mRNA (through conversion to cDNA), and at present, the technology has reached a level of sensitivity, accuracy, and practical ease, which has resulted in Q-PCR becoming a mainstream experimental technique. In the current study, we have used triplex Q-PCR to develop a fast, objective, and quantitative method for the detection of B16 melanoma metastases in the lungs. Since metastasis is the leading cause of melanoma-related deaths, the metastatic lesions represent the most important targets for potential new melanoma treatments, and hopefully our approach can be used to accelerate the process of pre-clinical evaluation of new therapies.

GP100 and TRP-2 are highly expressed in melanoma cells, except for when these have lost the ability to produce melanin. Using the relative expression of these two genes, we therefore were able to detect the presence of B16.F10gp melanoma cells in the lungs as early as 2 hours after i.v. challenge with at least 10^4^ cells. The actual detection limit of the assay, however, is likely to be lower than 10^4^ melanoma cells, as not all of the i.v. injected cells are expected to accumulate in the lungs. When comparing the gene expression of the singleplex, triplex, and one-step triplex Q-PCR, the expression pattern was found to be very similar regardless of the assay. This validates that all three Q-PCR types are sensitive methods for detecting melanoma lung metastasis, except in the case of amelanotic melanomas, and we decided to use the triplex Q-PCR for all subsequent experiments.

When analyzing the time-frame for tumor growth in the lungs post i.v. challenge with 10^5^ B16.F10gp cells, we found that the melanoma gene expression gradually decreased during the first 24 hours, followed by a marked increase of nearly 2 logs over the next 10 days. These data demonstrate a clear advantage of this method, the sensitivity of the Q-PCR. This allows for the detection of metastasis within hours after i.v. tumor exposure, and unlike visual inspection, the triplex Q-PCR makes it possible to objectively follow the kinetics of tumor cell growth, even during the early stages of tumor establishment.

It has long been known that NK cells represent a primary defense against tumor cell growth [17], [18]. After decades of research in the field, it is now known that NK cells detect and kill malignant cells with low MHC class I surface expression through the interaction of inhibitory and activating NK receptors [23], [24]. The tumorstatic activity of NK cells is mediated by different mechanisms including cell killing through the granule exocytosis pathway, Fas and/or TRAIL signalling, as well as the secretion of IFNγ [25]. For this reason, we speculated that NK cells might play a significant role in the decrease in tumor load observed in the lungs during the early phase following tumor inoculation. We therefore looked into the early melanoma gene expression after depletion of NK cells or infection with VSV, a known inducer of type I IFNs and NK cell activation [19], [20]. Infecting mice with VSV was found to markedly reduce the gene expression in the lungs, and this reduction was reversed by treatment with NK cell depleting antibodies. In contrast, little increase in melanoma gene expression was observed when NK depleting antibodies were used alone in uninfected mice. The contradicting nature of these results could be accounted for if the NK1.1 depleting antibodies are primarily successful in depleting the circulating pool of NK cells, but fail to penetrate into the lungs. Since the reduction of melanoma gene expression during VSV-induced inflammation is likely to be mediated predominantly by activated NK cells, which are newly recruited from the circulation, this could explain how NK cell depletion can eliminate the VSV-associated reduced gene expression. On the other hand, in uninfected mice sufficient resident lung NK cells may remain to control the early phase of tumor metastasis. However, another explanation could simply be that resting NK cells play no critical role in controlling initial tumor establishment during steady-state, but that activated NK cells, as found during VSV-induced inflammation, may reduce tumor metastasis. In the latter case, the early reduction of melanoma gene expression in naïve mice may simply reflect the gradual release of tumor cells transiently caught in the capillary bed of the lungs following tail vein inoculation.

Knowing that IFNγ and perforin play an important role in NK-mediated tumor control, we next sought to investigate the role of these mediators in tumor control during the early phase of lung metastasis. No difference in melanoma gene expression was observed between uninfected WT and IFNγ and/or perforin deficient mice. In contrast, the VSV-associated reduction of gene expression in WT mice was not observed in VSV-infected IFNγ/perforin deficient mice. In conclusion, these results suggest that in the steady-state situation, NK cells play only a minor role in the early reduction of the tumor load in the lungs, whereas NK cell-derived IFNγ and/or perforin seem to play an important role in the early tumor control in the lungs, when inflammation has induced an increased number of activated NK cells.

Vaccination against cancer represents a major challenge and at present, it has only been met with limited success. We have previously used an adenoviral vaccination approach, which was found to induce quite effective protection against solid B16.F10gp melanomas [13]. However, the real target for any melanoma vaccine is the metastases, and we therefore decided to test the anti-metastasis effects of the vaccines using the triplex Q-PCR method.

Due to the clear-cut anti-tumor effect of the vaccines against solid tumors, we predicted that the vaccines should be able to reduce the level of melanoma gene expression in the lungs, thus representing an alternative way of validating the Q-PCR method. We found no detectable difference in gene expression between vaccinated and non-vaccinated mice when tumor cells were inoculated 11 days post vaccination and the lungs removed 2 hours later. Since activation of the adaptive T cell response previously has been demonstrated to peak around day 10–14 post vaccination with Ad-Ii-GP or Ad-GP, it was somewhat unexpected to find that previous vaccination did not cause any difference in gene expression under these conditions [12], [21]. However, this probably reflects that it takes some time for the vaccine-induced antigen-specific T cells to accumulate in sufficient numbers in the lungs even at the height of the immune response. Thus, if the lungs were isolated from similarly treated mice 11 days after tumor inoculation, a clear-cut difference in the gene expression was found when comparing vaccinated and non-vaccinated mice. This capacity to control tumor gene expression was maintained for at least a month and probably longer after vaccination. Finally, we found that if mice were challenged at the peak of the primary CD8 T cell response, no more than 4 days were required to demonstrate a distinct difference between vaccinated and unvaccinated mice. Taken together, these results imply that prophylactic vaccination with Ad-GP or Ad-Ii-GP, in particular, can protect against melanoma metastasis to the lungs.

Direct comparison of our Q-PCR method to conventional analysis involving counting of metastatic foci under a dissection microscope revealed that tumor control could be detected earlier by our new method. Thus, metastatic foci could not be counted with certainty until day 5–6 after challenge, while a reduction in tumor gene expression may already be observed by day 4. More importantly, as it could be argued that reduction of tumor gene expression might not reflect reduced tumor growth, we would like to stress that parallel results were obtained when tumor metastasis was evaluated under the same conditions using the two different methodologies. Perhaps the most important difference between the two assays lies in the fact that, while visual inspection only considers numbers of metastatic foci regardless of size, the Q-PCR approach takes into account not only numbers of foci, but also numbers of cells per metastatic focus, thus delivering a more quantitative representation of tumor cell growth. This difference may be of particular relevance in situations where the antitumor effect under investigation is predominantly exerted at a late stage after metastasis rather than targeting the metastatic tumor cell itself.

In clinical settings, surgical removal of primary tumors has been demonstrated to increase the risk of tumor metastasis, since it can result in the release of tumor cells into the circulation [26]–[29]. It has also been found that surgery may promote metastasis indirectly by inducing growth of pre-established micrometastasis, through the reduction of anti-angiogenic factors, through the increase of released growth factors, as well as by the induction of immunosuppression caused by the medical procedure or perioperative stress and anxiety [26]. Trying to mimic the situation of vaccination simultaneously with surgically induced metastasis, mice were vaccinated and tumor challenged on the same day, and lungs were isolated 11 days later. Interestingly, we found a clear difference in melanoma gene expression between vaccinated and non-vaccinated mice. In addition, Ad-Ii-GP vaccinated mice showed improved protection against metastasis compared to Ad-GP. These results suggest that, in a clinical setting, vaccination with a vector such as Ad-Ii-GP concurrent with surgical removal of the primary tumor may substantially reduce the likelihood of metastatic spread caused by the surgery.

Finally, previous studies have shown that the Ad-Ii-GP-induced control of solid s.c. B16.F10gp tumors is dependent on CD8 T cells and IFNγ acting predominantly in a direct fashion on the tumor cells [13]. Using the triplex Q-PCR, we examined whether the same mechanisms were important for the vaccine-induced control of B16.F10gp lung metastasis. As in the case of s.c. tumors, we found that the control of B16.F10gp lung metastasis caused by Ad-Ii-GP vaccination was mediated through CD8 T cells and IFNγ, but not perforin. Thus, overall, our results indicate that the triplex Q-PCR represents a new and efficient method to quantify the level of B16 tumor metastasis.

## Supporting Information

Figure S1 **Standard curves and amplification plots for TRP-2, GP100, and GAPDH.** Samples were run as 10-fold dilution series of cDNA by singleplex (A), triplex (B), or one-step triplex (C) Q-PCR. All samples were run in duplicates. Efficiencies are close to 100% and the RSq is close to 1. Fluorescent dyes: GAPDH-Cy5 (purple), GP100-HEX (green), and TRP-2-FAM (blue).
(PDF)
