# Supplementary material for: Quantification of B16 Melanoma Cells in Lungs Using Triplex Q-PCR - A New Approach to Evaluate Melanoma Cell Metastasis and Tumor Control
Source: PLoS One. 2014 Jan 31;9(1):e87831. doi: 10.1371/journal.pone.0087831 (PMC3909236; doi:10.1371/journal.pone.0087831)
Supplement: Figure S1 — Standard curves and amplification plots for TRP-2, GP100, and GAPDH. Samples were run as 10-fold dilution series of cDNA by singleplex (A), triplex (B), or one-step triplex (C) Q-PCR. All samples were run in duplicates. Efficiencies are close to 100% and the RSq is close to 1. Fluorescent dyes: GAPDH-Cy5 (purple), GP100-HEX (green), and TRP-2-FAM (blue). (PDF) [file pone.0087831.s001.pdf]

Figure S1A

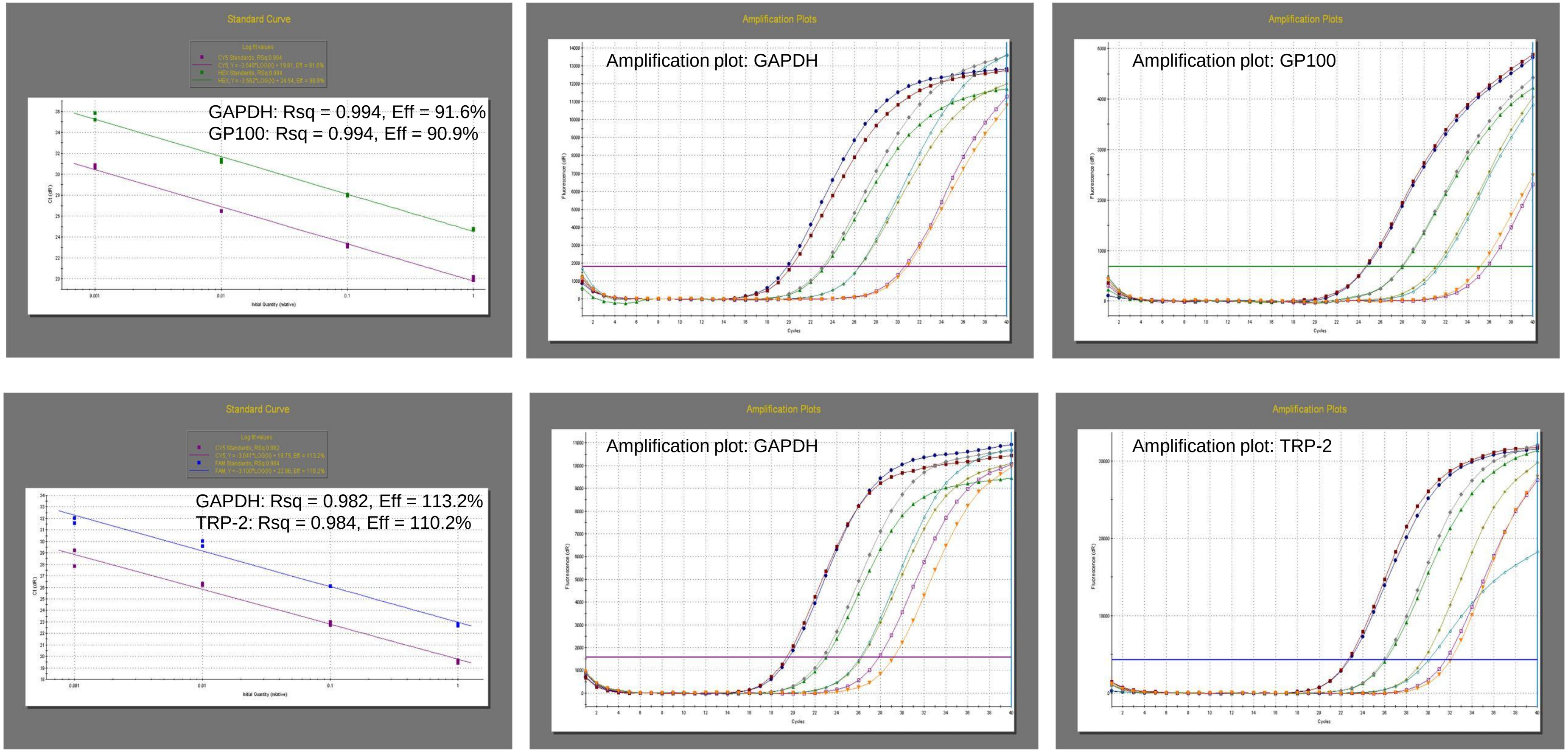

Standard Curve
GAPDH: Rsq = 0.994, Eff = 91.6%
GP100: Rsq = 0.994, Eff = 90.9%
Initial Quantity (relative)
Amplification Plots
Amplification plot: GAPDH
Cycles
Fluorescence (dR)
Amplification Plots
Amplification plot: GP100
Standard Curve
GAPDH: Rsq = 0.982, Eff = 113.2%
TRP-2: Rsq = 0.984, Eff = 110.2%
Amplification Plots
Amplification plot: GAPDH
Amplification Plots
Amplification plot: TRP-2

Figure S1B

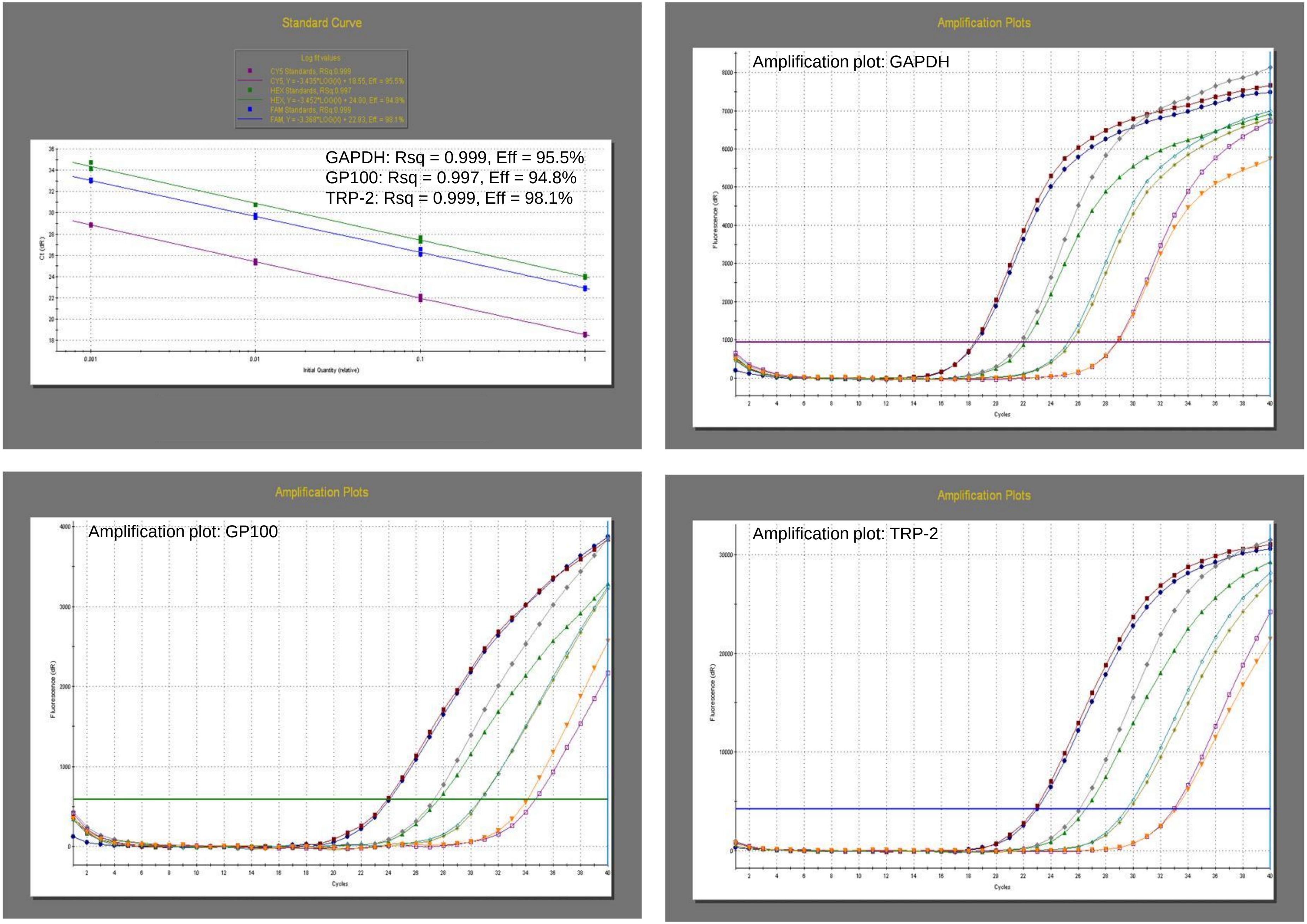
Standard Curve
GAPDH: Rsq = 0.999, Eff = 95.5%
GP100: Rsq = 0.997, Eff = 94.8%
TRP-2: Rsq = 0.999, Eff = 98.1%
Initial Quantity (relative)
Amplification Plots
Amplification plot: GAPDH
Fluorescence (dR)
Cycles
Amplification Plots
Amplification plot: GP100
Fluorescence (dR)
Cycles
Amplification Plots
Amplification plot: TRP-2
Fluorescence (dR)
Cycles

Figure S1C

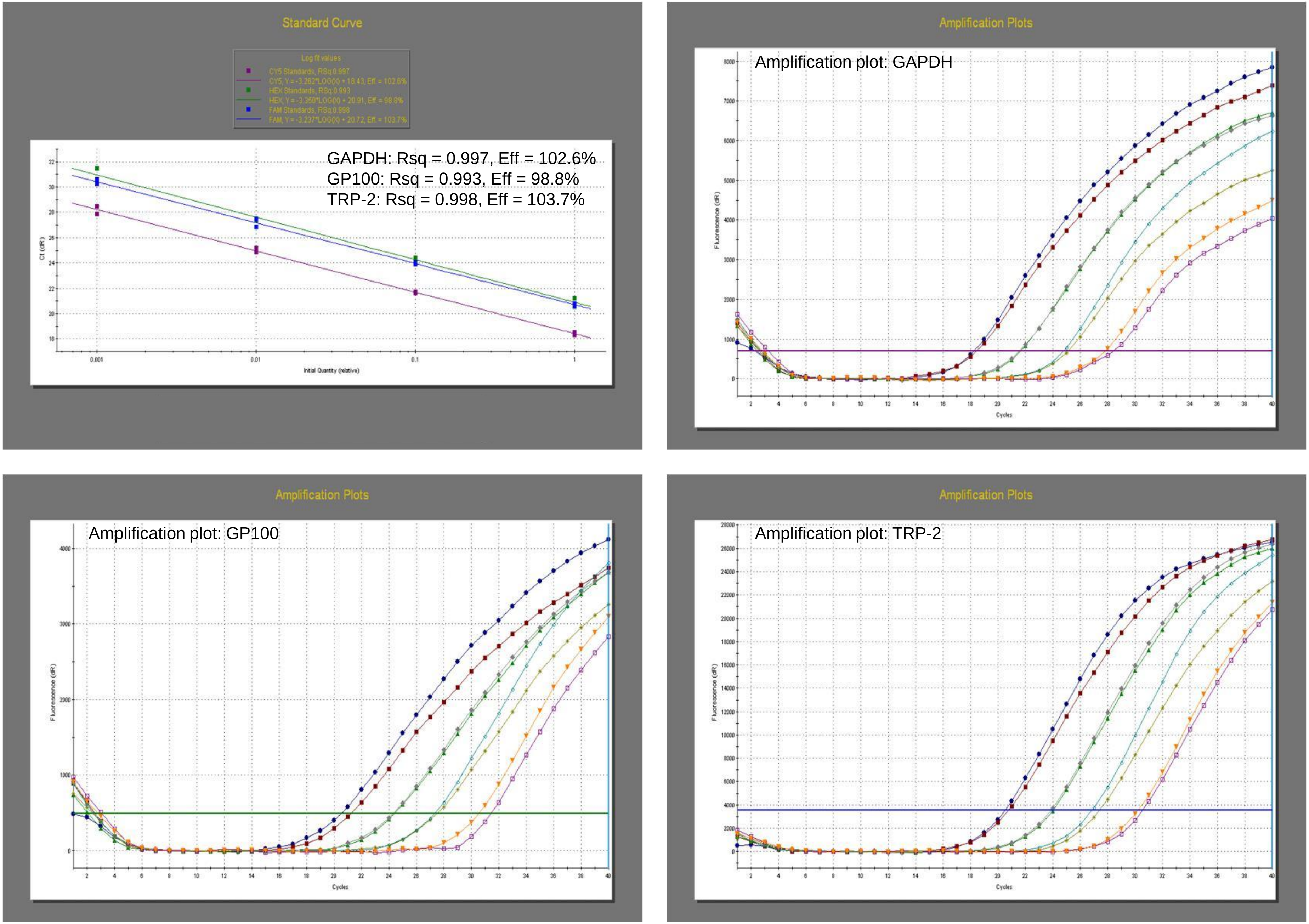
Standard Curve
Log fit values
CY5 Standards, RSq:0.997
CY5, Y = -3.262*LOG(X) + 18.43, Eff = 102.6%
HEX Standards, RSq:0.993
HEX, Y = -3.350*LOG(X) + 20.91, Eff = 98.8%
FAM Standards, RSq:0.998
FAM, Y = -3.237*LOG(X) + 20.72, Eff = 103.7%
GAPDH: Rsq = 0.997, Eff = 102.6%
GP100: Rsq = 0.993, Eff = 98.8%
TRP-2: Rsq = 0.998, Eff = 103.7%
Ct (dR)
Initial Quantity (relative)
Amplification Plots
Amplification plot: GAPDH
Fluorescence (dR)
Cycles
Amplification Plots
Amplification plot: GP100
Fluorescence (dR)
Cycles
Amplification Plots
Amplification plot: TRP-2
Fluorescence (dR)
Cycles
